# Dynamic cortical participation during bilateral, cyclical ankle movements: effects of aging

**DOI:** 10.1038/srep44658

**Published:** 2017-03-16

**Authors:** Takashi Yoshida, Kei Masani, Karl Zabjek, Robert Chen, Milos R. Popovic

**Affiliations:** 1 Rehabilitation Engineering Laboratory, Toronto Rehabilitation Institute, University Health Network, Toronto, Canada; 2 Institute of Biomaterials and Biomedical Engineering, University of Toronto, Toronto, Canada; 3 Department of Physical Therapy, University of Toronto, Toronto, Canada; 4 Division of Neurology, Department of Medicine, University of Toronto, Toronto, Canada; 5 Krembil Research Institute, University Health Network, Toronto, Canada

## Abstract

The precise role of the human primary motor cortex in walking is unknown. Our previous study showed that the primary motor cortex may contribute to specific requirements of walking (i.e., maintaining a constant movement frequency and bilaterally coordinating the feet). Because aging can impair (*i*) the ability to fulfill the aforementioned requirements and (*ii*) corticomuscular communication, we hypothesized that aging would impair the motoneuronal recruitment by the primary motor cortex during bilateral cyclical movements. Here, we used corticomuscular coherence (i.e., coherence between the primary motor cortex and the active muscles) to examine whether corticomuscular communication is affected in older individuals during cyclical movements that shared some functional requirements with walking. Fifteen young men and 9 older men performed cyclical, anti-phasic dorsiflexion and plantarflexion of the feet while seated. Coherence between the midline primary motor cortex and contracting leg muscles cyclically increased in both age groups. However, the coherence of older participants was characterized by (*i*) lower magnitude and (*ii*) mediolaterally broader and more rostrally centered cortical distributions. These characteristics suggest that aging changes how the primary motor cortex participates in the cyclical movements, and such change may extend to walking.

In humans, the primary motor cortex participates in the control of the basic patterns of walking^1^,^2^,^3^,^4^. However, the precise nature of its participation is not yet known. In our previous study (unpublished), we had observed that coherence between the midline primary motor cortex and the active leg muscles (i.e., corticomuscular coherence) increased dynamically during bilateral cyclical ankle movements. This finding suggested that the primary motor cortex contributed to maintaining a constant cyclical movement frequency and bilaterally coordinating the feet: functional requirements that are also present in walking.

Aging is associated with deficits in meeting the above requirements (i.e., increased movement variability and impaired bilateral coordination) during walking and other bilateral cyclical movements^5^,^6^,^7^,^8^. Aging is also associated with neuromuscular changes that can impair corticomuscular communication. These changes include decrease in the gray matter volume of the primary motor cortex^9^,^10^,^11^,^12^,^13^; decrease in the white matter volume of the posterior limbs of the internal capsule^13^,^14^, which contain the corticospinal tracts^15^,^16^; decrease in the number of motor neurons^17^,^18^,^19^; structural abnormalities of the neuromuscular junctions^20^; and re-organization of motor units that results in more fibers per neuron^21^,^22^,^23^. Indeed, previous studies have reported age-related reduction in (*i*) the amplitude of motor evoked potentials (i.e., corticospinal excitability)^24^ and (*ii*) corticomuscular coherence during sustained contractions of upper limb muscles^25^,^26^.

The purpose of this study was to examine how aging affected corticomuscular communication during movements that shared specific functional requirements with walking (i.e., bilateral cyclical ankle movements). In this study, corticomuscular communication was quantified by corticomuscular coherence. Based on the age-related impairment of motor performance and alteration of corticomuscular communication, we hypothesized that aging would be associated with lower magnitudes of corticomuscular coherence. To our knowledge, no study has examined how aging affects corticomuscular coherence during cyclical, anti-phasic movements. Several studies have examined the effects of aging on corticomuscular coherence^25^,^26^,^27^,^28^, but these studies are limited to sustained contractions.

## Results

### Motor performance

On average, young participants completed 170 ± 8 and 171 ± 6 cycles of self-paced and externally-paced movements, respectively. Older participants completed 165 ± 10 and 170 ± 2 cycles of self-paced and externally-paced movements, respectively. Among the participants, the minimum number of movement cycles was 139. Thus, for each participant, the first 139 of the recorded cycles were used to calculate the ensemble average of corticomuscular coherence. The inter-run rests ranged from 61 to 154 seconds for young participants and 60 to 156 seconds for older participants.

Table 1 summarizes the kinematics of the ankle movements. The mean cycle duration was significantly affected by aging (*F*_1,87_ = 4.66, *p* = 0.336 × 10^−1^) and the type of pacing (*F*_1,87_ = 6.44, *p* = 0.129 × 10^−1^), but not by the side of the body (*F*_1,87_ = 1.54 × 10^−5^, *p* = 0.997). *Post hoc* analysis showed that the mean cycle duration was significantly longer for older participants with self-pacing, compared to young participants with external pacing. The standard deviation of cycle durations was significantly affected by the type of pacing (*F*_1,87_ = 7.73, *p* = 0.666 × 10^−2^), but not by aging (*F*_1,87_ = 1.67, *p* = 0.200) or the side of the body (*F*_1,87_ = 2.86, *p* = 0.945 × 10^−1^). *Post hoc* analysis showed that, for both groups, the standard deviation was significantly greater with self-pacing. On the mean range of motion, the effects of aging (*F*_1,87_ = 3.90, *p* = 0.516 × 10^−1^), type of pacing (*F*_1,87_ = 0.0255, *p* = 0.873), and side of the body (*F*_1,87_ = 0.107, *p* = 0.744) were insignificant. On the standard deviation of the range of motion, the effects of aging (*F*_1,87_ = 0.312, *p* = 0.578), type of pacing (*F*_1,87_ = 1.63, *p* = 0.206), and side of the body (*F*_1,87_ = 0.253, *p* = 0.616) were also insignificant. The mean relative phase (*x* of *ϕ* in Table 1), which indicated the bilateral coordination of limbs, was significantly affected by aging (*F*_1,43_ = 4.24, *p* = 0.0456) but not by the type of pacing (*F*_1,43_ = 0.282, *p* = 0.598). *Post hoc* analysis showed that, although the movements were asymmetrical for both groups (with left dorsiflexion occurring slightly earlier than it should), young participants showed greater asymmetry than older participants. The standard deviation of the relative phase (*s* of *ϕ* in Table 1) was not significantly affected by aging (*F*_1,43_ = 1.19, *p* = 0.281) or the type of pacing (*F*_1,43_ = 0.537, *p* = 0.468). None of the parameters of motor performance was associated with a significant interaction between the factors (Supplementary Table S1).

To examine the effects of motion artifacts due to head movements, we quantified the cyclical linear movements of the markers at the electroencephalographic (EEG) electrode locations, AF_7_ and AF_8_. For both groups of participants, regardless of the type of pacing, the markers were within a volume of approximately 1 cm^3^ during each movement cycle. For young participants, the average cyclical linear head movements were no more than 7 mm, 6 mm, and 4 mm, in the rostrocaudal, mediolateral, and longitudinal directions, respectively. This was true for both self-paced and externally-paced movements. The equivalent measures for the older participants were no more than 8 mm, 7 mm, and 4 mm. Because the head movements were small, we assumed that the effects of motion artifacts due to head movements on EEG signals were negligible.

### Cyclical patterns of corticomuscular coherence

**Figure 1:**
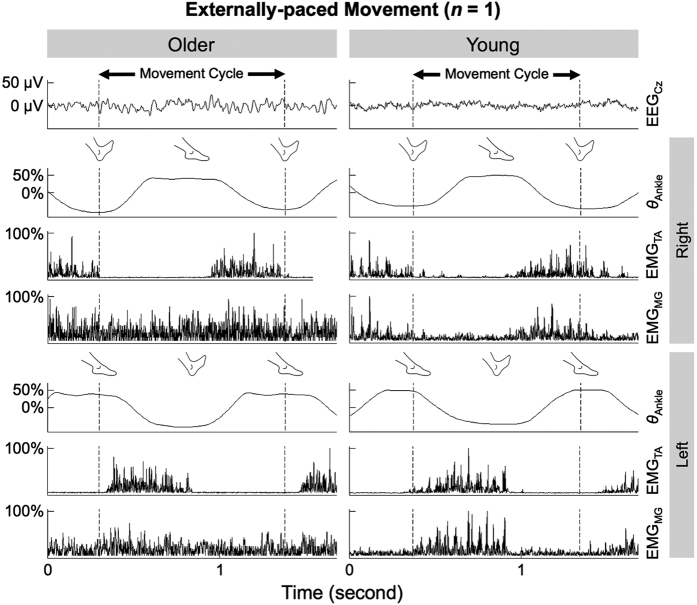
Ankle angles (*θ*_Ankle_), full-wave rectified EMG signals from the tibialis anterior (TA) and medial gastrocnemius (MG) muscles, and noise-reduced EEG signal from C_z_ of representative older and young participants during externally-paced ankle movements. Ankle angles have been centered and normalized to its range. EMG signals have also been normalized to its range. For the older participant, the maximum values of the shown EMG signals were 0.482 and 0.277 mV for the right and left TA muscles, respectively, and 0.0112 and 0.0138 mV for the right and left MG muscles, respectively. For the young participant, the maximum values of the shown EMG signals were 0.738 and 1.26 mV for the right and left TA muscles, respectively, and 0.0512 and 0.0569 mV for the right and left MG muscles, respectively.

Figure 1 shows brief time courses of all collected signals from representative young and older participants during externally-paced movements. During dorsiflexion, both participants showed increased activation of the tibialis anterior muscle, with no obvious discrepancy in the electroencephalographic (EMG) patterns. However, the young participant also showed co-contractions of the medial gastrocnemius muscles during dorsiflexion while such co-contraction was indiscernible in the older participant (Fig. 1). The above observations were also true for self-paced movements (Supplementary Fig. S1).

**Figure 2:**
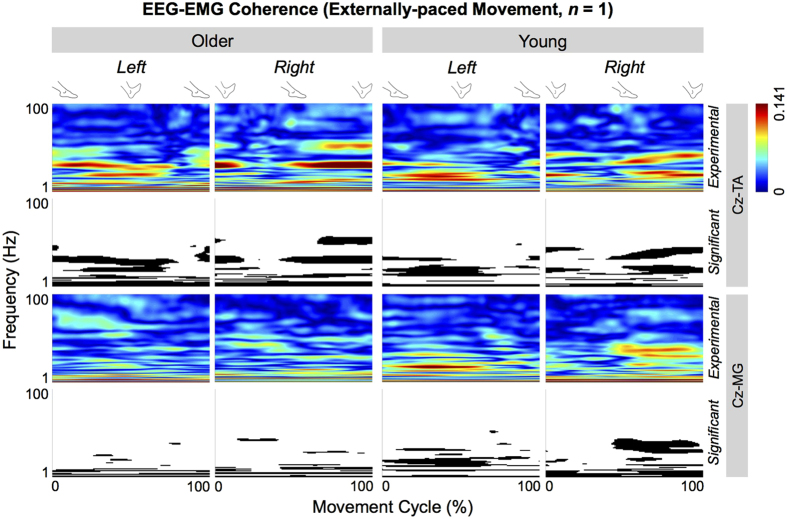
Cyclical corticomuscular coherence of representative older and young participants during externally-paced movements. Coherence is calculated between C_z_ and the tibialis anterior (TA) and medial gastrocnemius (MG) muscles. For each muscle, the black and white patterns in the bottom row indicate the significant portions of the patterns in the top row.

Figure 2 shows the cyclical corticomuscular coherence of the representative young and older participants during externally-paced movements. For both participants, the coherence between C_z_ and the tibialis anterior muscles increased cyclically, approximately coinciding with dorsiflexion (cf. Fig. 1). Furthermore, the cyclical increase occurred below 50 Hz, particularly between β to low-γ range, and exceeded the threshold of significance. The two participants differed in their coherence between C_z_ and the medial gastrocnemius muscles: the young participant showed a cyclical increase in coherence during dorsiflexion while the older participant showed no such pattern (Fig. 2). The above observations were also true for the self-paced movements (Supplementary Fig. S2).

### Validating corticomuscular coherence

**Figure 3:**
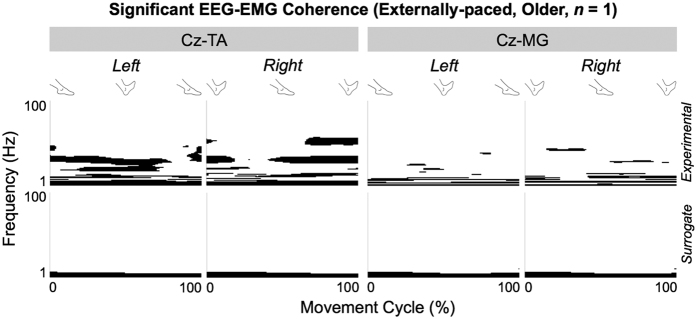
Significant portions of experimental and surrogate corticomuscular coherence for a representative older participant during externally-paced movements. Coherence is shown between C_z_ and the tibialis anterior (TA) and medial gastrocnemius (MG) muscles.

Figure 3 shows the significant portions of experimental and surrogate corticomuscular coherence for a representative older participant during externally-paced movements. Unlike experimental coherence, surrogate coherence did not show a cyclical increase at higher frequencies. Thus, shuffling the pairing between EEG and EMG signals abolished the cyclical increase in their coherence. However, shuffled pairing did not abolish their coherence at lower frequencies, typically below 6 Hz (Fig. 3). According to 2-way analysis of variance (ANOVA), the volume of significant coherence was significantly affected by the shuffled pairing above (*F*_1,372_ = 70.2, *p* = 0.112 × 10^−14^) and below 6 Hz (*F*_1,372_ = 40.2, *p* = 0.674 × 10^−9^). *Post hoc* analysis revealed that, after shuffling, the volume of significant coherence was (*i*) significantly smaller (and almost negligible) above 6 Hz and (*ii*) significantly larger below 6 Hz. Above 6 Hz, the volume of significant coherence was also significantly affected by aging (*F*_1,372_ = 4.86, *p* = 0.281 × 10^−1^), and *post-hoc* analysis revealed that the volume was significantly larger for young participants. Furthermore, above 6 Hz, aging and the type of coherence (i.e., surrogate or experimental) interacted significantly (*F*_1,372_ = 4.58, *p* = 0.329 × 10^−1^), probably indicating that young participants experienced greater reductions in the volume of significant coherence due to the shuffled pairing. Below 6 Hz, the volume of significant coherence was not significantly affected by aging (*F*_1,372_ = 0.0922, *p* = 0.762), and aging and the type of pacing did not interact significantly (*F*_1,372_ = 1.26, *p* = 0.262).

### Magnitude and frequency of corticomuscular coherence

**Figure 4:**
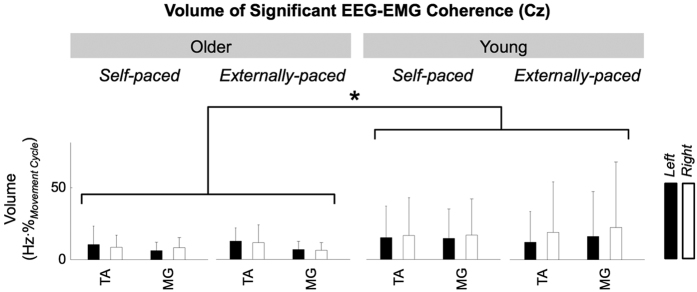
Volume of significant corticomuscular coherence between C_z_ and the tibialis anterior (TA) and medial gastrocnemius (MG) muscles. The error bars indicate inter-individual standard deviations.

For all participants, the threshold of significance was 0.0847. The magnitude of significant coherence was significantly affected by aging (*F*_1,177_ = 4.72, *p* = 0.311 × 10^−1^), and *post hoc* analysis revealed that the magnitude was smaller for older participants (Fig. 4). The magnitude was not significantly affected by the type of pacing (*F*_1,177_ = 0.113, *p* = 0.737), muscle (*F*_1,177_ = 0.0815, *p* = 0.776), or side of the body (*F*_1,177_ = 0.286, *p* = 0.593).

For young participants, the center frequency of significant coherence during externally-paced movements were 20.3 ± 4.2 and 20.5 ± 5.5 Hz for the left and right tibialis anterior muscles, respectively, and 20.7 ± 7.3 and 20.1 ± 4.0 Hz for the left and right medial gastrocnemius muscles, respectively. The equivalent values during self-paced movements were 18.1 ± 3.7 and 17.0 ± 5.1 Hz for the left and right tibialis anterior muscles, respectively, and 18.3 ± 4.1 and 18.7 ± 5.2 Hz for the left and right medial gastrocnemius muscles, respectively. For older participants, the center frequency of significant coherence during externally-paced movements were 19.9 ± 3.2 and 20.3 ± 4.1 Hz for the left and right tibialis anterior muscles, respectively, and 18.2 ± 5.5 and 20.3 ± 4.1 Hz for the left and right medial gastrocnemius muscles, respectively. The equivalent values during self-paced movements were 17.3 ± 3.5 and 19.0 ± 3.9 Hz for the left and right tibialis anterior muscles, respectively, and 19.6 ± 6.8 and 18.8 ± 4.0 Hz for the left and right medial gastrocnemius muscles, respectively. The frequency was not significantly affected by aging (*F*_1,177_ = 0.800, *p* = 0.372), type of pacing (*F*_1,177_ = 2.97, *p* = 0.866 × 10^−1^), muscle (*F*_1,177_ = 0.175, *p* = 0.676), or side of the body (*F*_1,177_ = 0.00747, *p* = 0.931). For neither the magnitude nor the frequency, did the factors of 4-way ANOVA interact significantly (Supplementary Table S2).

### Cortical distribution of corticomuscular coherence

**Figure 5:**
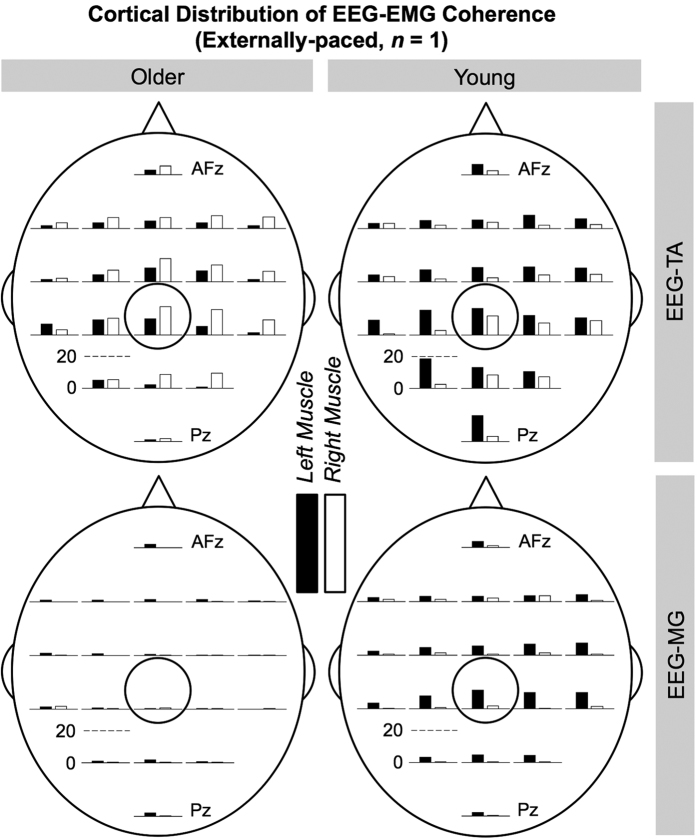
Cortical distributions of significant coherence between EEG signals and EMG signals from the tibialis anterior (TA) and medial gastrocnemius (MG) muscles of representative older and young participants during externally-paced movements. C_z_ is circled. At each electrode location, the bar indicates the volume of significant coherence, measured in Hz multiplied by the percentage of movement cycle (Hz%_*Movement Cycle*_). The scale of the vertical axis is the same for all distributions.

Figure 5 shows the cortical distributions of significant coherence for representative young and older participants during externally-paced movements. The representative young participant generally showed cortical distributions that centered around C_z_ for both muscles. The representative older participant also showed such distributions for the tibialis anterior muscles, but not for the medial gastrocnemius muscles (Fig. 5). These observations were also true for self-paced movements (Supplementary Fig. S3).

Tables 2 and 3 summarize the results of fitting a bivariate normal distribution to the cortical distributions of significant coherence for older and young participants, respectively. Supplementary Table S3 and Supplementary Table S4 summarize the results of applying 4-way ANOVA on the parameters in Tables 2 and 3. For optimally fitted normal distributions, the root-mean square deviation (RMSD), coefficient of determination (COD), and peak value (*A*) were not significantly affected by any of the factors: aging, type of pacing, muscle, or side of the body (Supplementary Table S3).

The standard deviation in the rostrocaudal direction (*σ*_RC_) was also not significantly affected by any of the factors, but the standard deviation in the mediolateral direction (*σ*_ML_) was significantly affected by aging (*F*_1,113_ = 4.03, *p* = 0.0471). *Post hoc* analysis showed that *σ*_ML_ was slightly but significantly smaller for younger participants. The mean of the optimally fitted normal distributions in the rostrocaudal direction (*μ*_RC_) was significantly affected by aging (*F*_1,113_ = 4.63, *p* = 0.0335) and muscle (*F*_1,113_ = 4.75, *p* = 0.0314). *Post hoc* analysis showed that the fitted normal distributions were located more rostrally for older participant and the medial gastrocnemius muscles. The mean of the optimally fitted normal distributions in the mediolateral direction (*μ*_MC_) was not significantly affected by any of the factors. For *σ*_RC_, the type of pacing and muscle interacted significantly (F_1,113_ = 6.27, *p* = 0.0137). This interaction probably indicated that, with external pacing, *σ*_RC_ tended to increase for the tibialis anterior muscles and decrease for the medial gastrocnemius muscles. For other parameters in Tables 2 and 3, the factors of the 4-way ANOVA did not interact significantly (Supplementary Table S4).

## Discussion

Between young and older participants, we observed discrepancies in several aspects of corticomuscular coherence during bilateral cyclical ankle movements. As we hypothesized, the magnitude of cyclical corticomuscular coherence was lower for older participants than for younger participants (Fig. 4). The lower magnitude of coherence suggests that the primary motor cortex participates differently in older individuals during simple cyclical movements: the participation is either (*i*) less overall or (*ii*) less linear, as coherence only quantifies the liner aspect of corticomuscular communication.

Our result agreed with previous studies that observed decreased magnitude of corticomuscular coherence in older individuals during sustained contractions of upper limb muscles^25^,^26^. However, during sustained contractions of upper limb muscles, some studies reported age-related increase in the magnitude of coherence^27^,^28^. Kamp *et al*.^28^ had attributed the age-related increase to greater cortical involvement that occurred as compensation against the effects of aging. Although such phenomenon complies with the compensation hypothesis^29^, it may be task-specific to sustained isometric contraction with continuous visual monitoring of the level of muscle activation^27^,^28^. With greater conscious control of muscle activation than what the ankle movements required in this study, sustained contractions are more likely to depend on the primary motor cortex to directly activate the muscles. In this case, compensation by increasing the cortical involvement is plausible. Furthermore, the ankle movements in this study may have also depended on subcortical and spinal neuronal networks to generate the cyclical movements. If so, age-related increase in activity could have occurred outside the corticomuscular communication.

Unlike the magnitude of corticomuscular coherence, the parameters of motor performance did not indicate known age-related deteriorations (i.e., increased movement variability and impaired bilateral coordination). Thus, despite slower movements with self-pacing, the ability to perform the ankle movements was generally preserved among older participants (Table 1).

Despite the lack of age-related discrepancy in motor performance, aging affected the magnitude of corticomuscular coherence. This finding suggests several possibilities: (*i*) the cyclical increase in corticomuscular coherence was irrelevant to the functional requirements of the ankle movements; (*ii*) the observed age-related discrepancy indicated pre-symptomatic changes in motor control, in a similar fashion to the pre-symptomatic pathology of neurodegenerative diseases such as Parkinson’s disease^30^; and (*iii*) there was a floor effect (i.e., magnitude of coherence would have shown greater age-related discrepancy had the task been more demanding). To confirm the functional relevance of corticomuscular coherence, future research should consider tasks that are either (*i*) challenging enough to induce a difference in performance between young and older individuals or (*ii*) varied in difficulty to examine how the coherence relates to difficulty (e.g., inclusion of in-phasic movements or multiple movement frequencies). Alternatively, future studies could target older individuals, whose performance of a particular movement is known to be impaired.

The cortical distribution of corticomuscular coherence was slightly but significantly broader in the mediolateral direction for older participants (indicated by *σ*_ML_ in Tables 2 and 3). Functional neuroimaging studies have shown that older individuals recruit additional cortical, subcortical, or cerebellar areas to perform various isolated movements of the fingers, wrists, and ankles^31^,^32^,^33^,^34^,^35^,^36^,^37^,^38^. If older participants engaged a broader area of the primary motor cortex, its activity could have propagated to EEG electrodes that abut C_z_ in the mediolateral directions, thereby broadening the cortical distribution of coherence. Aging was also associated with a more rostrally centered cortical distribution of corticomuscular coherence (illustrated in Fig. 5 and Supplementary Fig. S3). The sub-division of the primary motor cortex into rostral and caudal regions has been suggested by several studies^39^,^40^,^41^. For example, using retrograde transneuronal transmission of the rabies virus in rhesus monkeys, Rathelot and Strick^39^ have shown that approximately 70 to 90% of corticospinal neurons, which project monosynaptically to the motoneurons of proximal and distal forelimb muscles, were located in the caudal region of the primary motor cortex. The rostral region contained approximately 5 to 10% of such corticospinal neurons, with the remainder located in rostral region of the post-central gyrus^39^. It is possible that the caudal region of the primary motor cortex is more vulnerable to age-related deterioration, thereby causing older individuals to engage the rostral region. Similarly, Plow *et al*.^42^ have reported that the cortical distribution of motor evoked potentials is more rostrally centered for older individuals (i.e., the center of corticospinal excitability is shifted rostrally).

There were several limitations in this study. First, our older participants included two individuals aged 51 and 61 years, who are younger than individuals normally considered old (e.g., 65 years old). Also, without imaging, we could not determine whether the older participants had experienced neural changes that would affect corticomuscular communication. However, out of such neural changes, (*i*) the gray matter volume declines more or less steadily from the age of 20 years^9^,^10^,^11^,^12^,^13^, (*ii*) the white matter volume either starts to decline around the age of 40 years^13^ or declines steadily from the age of 20 years^14^, (*iii*) the number of motor neurons decreases steadily from the age of 20 years old^17^,^18^ though the decrease may accelerate around the age of 60 years^19^, and (*iv*) the apparent re-organization of the motor units occurs more or less steadily from the age of 20 years^21^,^22^. Furthermore, the reduction in corticospinal excitability^24^ and corticomuscular coherence^26^ has been observed in individuals older than 55 years of age. Therefore, despite the inclusion of two younger individuals, we assumed that our older participants had undergone at least some change in their corticomuscular communication. The second limitation is the uncertainty regarding the levels of muscle and central fatigue due to the ankle movements. Particularly, young participants may have experienced some muscle fatigue during the second run of externally-paced movements (i.e., the mean power frequency of the EMG signal from the right tibialis anterior muscle significantly decreased by 8.35 ± 7.81 Hz during the last three cycles, compared to the first three cycles), and the fatigue could have enhanced corticomuscular coherence^43^ though we suspect the effects to be modest^44^. Third, we did not consider a sedentary lifestyle as a significant confounder. Although the task that we chose for this study was not demanding in terms of power output, cardiopulmonary stress, precision, or complexity (i.e., the movements were unfamiliar to the participants yet simple enough to learn in a short amount of time and sustain for prolonged periods), a sedentary lifestyle could have affected corticomuscular communication independently from age-related changes. Fourth, the power of our statistical analysis may be limited due to the multi-factorial design and the small sample size. Lastly, coherence between surface EEG and EMG signals is only a gross measure of corticomuscular communication as a scalp EEG signal is the sum of all electrical field potentials in the vicinity of the measuring electrode, and a surface EMG signal is the sum of all motor unit action potentials in the vicinity of the measuring electrode.

In this study, young and older participants performed cyclical, anti-phasic ankle movements. During this movement, we observed discrepancies in the magnitude and cortical distributions of corticomuscular coherence between young and older participants. The coherence of older participants was characterized by (*i*) lower magnitude and (*ii*) mediolaterally broader and more rostrally centered cortical distributions. The lower magnitude suggests that the primary motor cortex either participates less in the control of the movement or in a less linear fashion (e.g., polysynaptically via spinal circuits). The broader and rostrally shifted cortical contributions may indicate compensation against age-related neuromuscular changes. Thus, we have shown that corticomuscular communication is affected in older individuals during bilateral cyclical movements, which share specific functional requirements with walking. Aging may similarly affect corticomuscular communication during walking.

## Methods

### Participants

By convenience sampling, we recruited 16 young men and 11 older men. One young participant and two older participants were excluded from data analysis because artifacts could not be removed sufficiently from their EEG signals. The remaining 15 young participants were 27 ± 7 years old, 177 ± 7 cm tall, and 75 ± 11 kg in weight. The remaining 9 older participants were 66 ± 7 years old, 176 ± 6 cm tall, and 86 ± 8 kg in weight. All participants were able to walk unassisted and reported no neurological disorders or dementia. Before participating in this study, all participants provided their written informed consent. All experimental protocols, which were performed according to the relevant guidelines, had been approved by the University Health Network Research Ethics Board, Toronto, Canada.

### Experimental task

Each participant performed 6 one-minute runs of cyclical ankle movements while sitting. The first run was always externally paced by the sound of a metronome, and subsequent runs alternated between self- and externally-paced movements. Between runs, the participants rested briefly. The self- and externally-paced conditions have been included because age-related discrepancies have been observed with both types of pacing^5^,^6^,^31^,^34^,^37^,^38^. During externally-paced runs, the participants were instructed to dorsiflex and plantarflex their feet in an anti-phasic manner: at each beat of the metronome, which had been set to 108 beats per minute, one foot was maximally dorsiflexed and the other foot was maximally plantarflexed. During self-paced runs, the participants were instructed to maintain the same rhythm as the externally-paced runs. Before the first run, the participants practiced the movement until they felt comfortable with the rhythm and anti-phasic coordination of the limbs. During each run, the participants were instructed to gaze forward and look at a bullseye. They were also instructed to relax their upper body and to refrain from moving their head, talking, swallowing, coughing, clenching their jaw, and excessively blinking. Given the simplicity of movement, we assumed that it was easy to retain the necessary motor skills during inter-run rests.

### Data collection

All signals were recorded in one-minute epochs. Each epoch (*i*) began several cycles after the participant had started the movement and (*ii*) ended after approximately one minute, before the participant was told to stop the movement. The sampling of kinematic data, EEG signals, and EMG signals were synchronized by an analogic switch.

To record kinematic data, we used an optical motion capture system. The system comprised a data acquisition device (MX Giganet, Vicon Motion Systems Ltd., United Kingdom), nine optical cameras (Bonita, Vicon Motion Systems Ltd., United Kingdom), data acquisition software (Nexus 1.8.5, Vicon Motion Systems Ltd., United Kingdom), and 14 mm retroreflective markers. The participants wore socks and a tight-fitting outfit. To track head movements, markers were placed over the EEG electrode locations, AF_7_ and AF_8_. To track lower body movements, markers were placed bilaterally over the greater trochanters, lateral epicondyles of the femur, lateral malleoli, and second metatarsal heads. The marker positions were sampled at 100 Hz.

To record EEG signals, we used an active electrode system (g.GAMMAsys, g.tec medical engineering GmbH, Austria) with signal amplifiers (g.USBamp, g.tec medical engineering GmbH, Austria) and recording software (g.Recorder, g.tec medical engineering GmbH, Austria). We used a monopolar montage to record EEG signals from 20 locations, which covered the midline primary motor cortex (C_z_) and its vicinity: AF_z_, F_z_, F_1_, F_2_, F_3_, F_4_, FC_z_, FC_1_, FC_2_, FC_3_, FC_4_, C_z_, C_1_, C_2_, C_3_, C_4_, CP_z_, CP_1_, CP_2_, and P_z_^45^. The reference electrode was placed on the left ear lobe and the ground electrode over the right zygomatic process of the temporal bone. The signals were sampled at 1.2 kHz without filtering.

To record EMG signals from the tibialis anterior muscle and the medial head of the gastrocnemius muscle on both sides, we measured the muscle activities using a wireless EMG system (Trigno™ Wireless EMG System, Delsys Inc., United States), which had a bandwidth of 20 to 450 Hz and the common mode rejection ratio of over 80 dB. All EMG signals were sampled at 2 kHz.

### Data analysis

All calculations were performed in a commercial numerical computing environment (MATLAB R2014b, The MathWorks, Inc., United States).

For each participant, we calculated three measures of performance: the range of motion at the ankle, movement cycle durations, and the relative phase between the left and right ankles. For each measure, the intra-individual mean and standard deviation were calculated across all movement cycles. Each cycle was defined by two consecutive local maxima in the vertical elevation of the motion-capture marker over the second metatarsal head of the right foot. The relative phase was calculated according to the method described by Abe *et al*.^46^. On the intra-individual mean and standard deviation of cycle durations and range of motion, we performed 3-way ANOVA with (*i*) aging (i.e., young or older), (*ii*) type of pacing (i.e., self- or externally-paced), and (*iii*) sides of the body (i.e., left or right) as factors. On the intra-individual mean and standard deviation of relative phase (with right dorsiflexion leading the cycle), we performed 2-way ANOVA with (*i*) aging and (*ii*) type of pacing as factors.

Using zero-phase digital filtering, the EEG signals were notch-filtered at 60 Hz and band-pass filtered between 0.5 and 100 Hz. Then, the filtered EEG signals were decomposed by independent component analysis^47^,^48^. According to the principles described by Libenson^49^, the filtered EEG signals and their independent components were visually inspected for artifacts. The contributions of independent components that contained an artifact were subtracted from the filtered EEG signals to produce noise-reduced EEG signals. This subtraction was restricted to the observed duration of the artifactual waveform. The EMG signals were centered and then full-wave rectified. In corticomuscular coherence, the assumption is that rectification would enhance the power spectral density of the EMG signal at the frequency of common input that recruits the constituent motor units. This assumption is supported by experimental evidence^50^ and computational modeling^51^.

For each participant, corticomuscular coherence was calculated between all EEG electrode locations and the two muscles on both sides. The noise-reduced EEG signals and rectified EMG signals were synchronized by down-sampling them at 400 Hz, and their wavelet coherence was calculated using the complex Morlet wavelet. The resultant corticomuscular coherence (i.e., approximately one-minute long) was segmented into individual movement cycles and ensemble-averaged to yield a pattern of corticomuscular coherence over one movement cycle).

For each participant, the magnitude of cyclical coherence was examined at all EEG electrode locations. Cyclical corticomuscular coherence was binned across frequency and time: binning across frequency resulted in one pixel per Hz between 1 and 100 Hz; binning across time resulted in one pixel per percent of the movement cycle. Then, above 6 Hz, we calculated the integral of coherence with all pixels that exceeded the threshold of significance (i.e., the volume of significant coherence). The volume of significant coherence was calculated in units of Hz multiplied by the percent of movement cycle duration (Hz%_*Movement Cycle*_). The threshold of significance was calculated for the range of 1 to 100 Hz, using the equation by Ushiyama *et al*.^52^. At the EEG electrode position, C_z_, we also calculated the center frequency (*f*_*c*_) for the volume of significant coherence (i.e., geometric centroid along frequency). On the volume and center frequency of significant coherence at C_z_, we performed 4-way ANOVA with (*i*) aging, (*ii*) type of pacing, (*iii*) side of the body, and *iv*) muscle (i.e., tibialis anterior or medial gastrocnemius muscles) as factors. All of these factors were relevant to aging and corticomuscular communication: both types of pacing have been associated with age-related discrepancies in motor performance and brain activation^5^,^6^,^31^,^34^,^37^,^38^, aging is known to affect bilateral coordination^6^,^7^,^8^, and corticospinal connection differs between the tibialis anterior and gastrocnemius muscles^53^. The number of movement cycles could affect the magnitude of coherence in an ensemble average. Therefore, for the above ANOVA, the ensemble average for each participant was calculated with the minimum number of cycles completed among the participants.

For each participant, a cortical distribution was formed with volumes of significant coherence between 13 and 30 Hz (i.e., the β band). These cortical distributions were quantified using surface fitting: we fitted a bivariate normal distribution to each participant’s cortical distribution of the volume of significant coherence. To compare the cortical distributions, 4-way ANOVA was performed on the following parameters of surface fitting: the mean (i.e., location of peak value) and standard deviation of the fitted normal distribution, root-mean-square deviation, and coefficient of determination. The factors of the 4-way ANOVA were (*i*) aging, (*ii*) type of pacing, (*iii*) side of the body, and (*iv*) muscle. The fitted distributions, whose coefficient of determination was below 0.5 or whose peak was located outside the studied cortical area, were excluded from the analysis.

If any factor in ANOVA showed a significant main effect, we performed *post hoc* analysis with Tukey’s honestly significant difference procedure. The significant level was set to 5% for all tests.

To validate the cyclical patterns of corticomuscular coherence, we calculated surrogate coherence at C_z_. For each participant, an ensemble average of coherence was calculated by pairing the *i*^th^ cycle of an EEG signal with the *j*^th^ cycle of an EMG signal, such that *i* ≠ *j* and none of the original pairing was preserved. For each participant, 100 such ensemble averages were calculated with differently permutated pairing of EEG and EMG signals, and the average magnitude of the 100 ensemble averages was used as surrogate coherence. In preliminary analysis, we observed that surrogate coherence generally showed relatively high magnitude below 6 Hz. Thus, we also examined how surrogate and experimental coherence at C_z_ differed in magnitude above and below 6 Hz. This comparison was performed by 2-way ANOVA with (*i*) aging and (*ii*) type of coherence as factors. The ANOVA was performed separately above and below 6 Hz.

## Additional Information

**How to cite this article**: Yoshida, T. *et al*. Dynamic cortical participation during bilateral, cyclical ankle movements: effects of aging. *Sci. Rep.* **7**, 44658; doi: 10.1038/srep44658 (2017).

**Publisher's note:** Springer Nature remains neutral with regard to jurisdictional claims in published maps and institutional affiliations.

## Supplementary Material

Supplementary Information

## Figures and Tables

**Table 1:** Kinematics of cyclical ankle movements for young and older participants.

Measurement			Self-paced Movements		Externally-paced Movements	
	Group		Left Ankle	Right Ankle	Left Ankle	Right Ankle
Cycle Duration (Seconds)	Young	*x*	1.11 ± 0.05	1.11 ± 0.05	1.10 ± 0.02	1.10 ± 0.02
		*s*	0.072 ± 0.019	0.0657 ± 0.0163	0.063 ± 0.014	0.0589 ± 0.0159
	Older	*x*	1.15 ± 0.08	1.15 ± 0.08	1.11 ± 0.00	1.11 ± 0.00
		*s*	0.0681 ± 0.0193	0.0636 ± 0.0167	0.0592 ± 0.0137	0.0512 ± 0.0108
Range of Motion (Degrees)	Young	*x*	38.2 ± 8.0	38.1 ± 5.8	37.8 ± 8.5	38.0 ± 5.8
		*s*	2.55 ± 1.09	2.62 ± 1.30	2.86 ± 0.99	2.68 ± 0.76
	Older	*x*	40.5 ± 9.2	41.3 ± 9.3	41.0 ± 8.3	42.2 ± 8.3
		*s*	2.58 ± 0.80	2.66 ± 0.50	3.16 ± 1.07	2.77 ± 1.00
*ϕ* (Degrees)	Young	*x*	185 ± 6	175 ± 6	185 ± 5	175 ± 5
		*s*	12.3 ± 3.9	12.7 ± 4.2	12.9 ± 4.3	13.1 ± 4.3
	Older	*x*	182 ± 6	178 ± 6	181 ± 5	179 ± 5
		*s*	10.7 ± 4.7	10.8 ± 4.1	11.6 ± 3.9	12.3 ± 3.6

Each entry shows the mean ± standard deviation among participants. *x* and *s* indicate the intra-participant mean and standard deviation, respectively. *ϕ* is the relative phase that indicates the bilateral coordination of the limbs (*ϕ* = 180° for symmetrical coordination).

**Table 2:** Parameters (Par.) of fitted bivariate normal distributions for older participants.

Par.	Self-paced Movement				Externally-paced Movement			
	TA		MG		TA		MG	
	Left	Right	Left	Right	Left	Right	Left	Right
*n*	6	5	5	6	9	5	5	2
RMSD	0.965 ± 0.794	1.29 ± 0.55	0.843 ± 0.636	1.18 ± 0.46	1.60 ± 1.47	1.37 ± 0.81	1.36 ± 1.24	0.757 ± 0.763
COD	0.758 ± 0.151	0.732 ± 0.117	0.703 ± 0.158	0.741 ± 0.142	0.666 ± 0.139	0.719 ± 0.117	0.751 ± 0.074	0.858 ± 0.044
*A*	8.53 ± 8.92	8.77 ± 6.42	5.95 ± 5.79	7.88 ± 4.78	8.65 ± 7.23	8.07 ± 6.53	7.41 ± 5.89	5.72 ± 5.27
*σ*_RC_	1.07 ± 0.33	1.33 ± 0.22	1.31 ± 0.23	1.38 ± 0.25	1.32 ± 0.36	1.25 ± 0.27	1.19 ± 0.17	1.15 ± 0.21
*σ*_ML_	1.44 ± 0.49	1.52 ± 0.29	1.83 ± 0.54	1.55 ± 0.46	1.51 ± 0.63	1.77 ± 0.62	2.18 ± 0.74	1.56 ± 0.20
*μ*_RC_	0.498 ± 0.791	0.587 ± 0.363	0.622 ± 0.541	0.506 ± 0.284	0.183 ± 0.671	0.468 ± 0.190	0.685 ± 0.357	0.736 ± 0.071
*μ*_ML_	0.151 ± 0.603	0.122 ± 0.355	0.0489 ± 0.6677	0.0531 ± 0.3943	-0.0552 ± 0.4156	0.743 ± 0.529	0.0115 ± 0.8911	0.0283 ± 0.590

Each entry is the mean ± standard deviation for *n* cortical distributions of coherence between EEG signals and EMG signals from tibialis anterior (TA) and medial gastrocnemius (MG) muscles. RMSD stands for root-mean-square deviation, and COD stands for coefficient of determination. *A, σ,* and *μ* are respectively the peak value, standard deviation, and mean of the fitted bivariate normal distributions. *A* is measured in Hz multiplied by the percentage of movement cycle. The mean is located on the rostrocaudal-mediolateral (*μ*_RC_, *μ*_ML_) coordinate system, where (0,0) indicates C_z_. A displacement by one on the coordinate system corresponds to a displacement by one electrode location in the rostrocaudal or mediolateral direction. Positive rostrocaudal and mediolateral coordinates respectively indicate anterior and left.

**Table 3:** Parameters (Par.) of fitted bivariate normal distributions for young participants.

Par.	Self-paced Movement				Externally-paced Movement			
	TA		MG		TA		MG	
	Left	Right	Left	Right	Left	Right	Left	Right
*n*	9	10	13	7	9	10	9	10
RMSD	1.46 ± 1.17	1.69 ± 2.01	1.27 ± 0.85	2.01 ± 1.35	2.37 ± 3.17	2.44 ± 4.61	1.96 ± 3.50	3.10 ± 6.40
COD	0.777 ± 0.122	0.808 ± 0.101	0.706 ± 0.146	0.742 ± 0.172	0.674 ± 0.125	0.770 ± 0.101	0.776 ± 0.140	0.671 ± 0.120
*A*	14.9 ± 21.2	16.2 ± 26.6	12.3 ± 19.2	23.3 ± 32.0	13.7 ± 22.8	18.3 ± 33.1	15.4 ± 29.0	21.3 ± 45.9
*σ*_RC_	1.10 ± 0.19	1.03 ± 0.29	1.21 ± 0.43	1.22 ± 0.33	1.33 ± 0.17	1.12 ± 0.30	1.01 ± 0.23	1.20 ± 0.234
*σ*_ML_	1.35 ± 0.38	1.34 ± 0.80	1.33 ± 0.43	1.60 ± 0.76	1.79 ± 0.51	1.18 ± 0.39	1.55 ± 0.55	1.55 ± 0.399
*μ*_RC_	0.0580 ± 0.3932	0.0136 ± 0.4688	0.265 ± 0.616	0.277 ± 0.516	0.400 ± 0.438	0.201 ± 0.658	0.516 ± 0.525	0.623 ± 0.738
*μ*_ML_	−2.86 × 10^−3^ ± 0.44	0.0194 ± 0.5592	0.0908 ± 0.7381	0.126 ± 0.577	−0.125 ± 0.383	0.0240 ± 0.5900	0.0790 ± 0.6149	0.340 ± 0.412

Each entry is the mean ± standard deviation for *n* cortical distributions of coherence between EEG signals and EMG signals from tibialis anterior (TA) and medial gastrocnemius (MG) muscles. RMSD stands for root-mean-square deviation, and COD stands for coefficient of determination. *A, σ,* and *μ* are respectively the peak value, standard deviation, and mean of the fitted bivariate normal distributions. *A* is measured in Hz multiplied by the percentage of movement cycle. The mean is located on the rostrocaudal-mediolateral (*μ*_RC_, *μ*_ML_) coordinate system, where (0,0) indicates C_z_. A displacement by one on the coordinate system corresponds to a displacement by one electrode location in the rostrocaudal or mediolateral direction. Positive rostrocaudal and mediolateral coordinates respectively indicate anterior and left.
