# Supplementary material for: Dynamic cortical participation during bilateral, cyclical ankle movements: effects of aging
Source: Sci Rep. 2017 Mar 16;7:44658. doi: 10.1038/srep44658 (PMC5353607; doi:10.1038/srep44658)
Supplement: Supplementary Information [file srep44658-s1.doc]

**Supplementary Information**

**Title:** Dynamic cortical participation during bilateral, cyclical ankle movements: effects of aging

**Authors:** Takashi Yoshida^a,b^, ^*^Kei Masani^a,b^, Karl Zabjek^c^, Robert Chen^d,e^, and Milos R. Popovic^a,b^

**Institutional affiliations:**

1. Rehabilitation Engineering Laboratory, Toronto Rehabilitation Institute, University Health Network, Toronto, Canada
2. Institute of Biomaterials and Biomedical Engineering, University of Toronto, Toronto, Canada
3. Department of Physical Therapy, University of Toronto, Toronto, Canada
4. Division of Neurology, Department of Medicine, University of Toronto, Toronto, Canada
5. Krembil Research Institute, University Health Network, Toronto, Canada

# Supplementary FigureS

**Supplementary Figure S1. Ankle angles (*θ*_Ankle_), full-wave rectified EMG signals from the tibialis anterior (TA) and medial gastrocnemius (MG) muscles, and noise-reduced EEG signal from C_z_ of representative older and young participants during self-paced ankle movements.** Ankle angles have been centered and normalized to its range. EMG signals have also been normalized to its range. For the older participant, the maximum values of the shown EMG signals were 0.406 and 0.245 mV for the right and left TA muscles, respectively, and 0.0988 and 0.0100 mV for the right and left MG muscles, respectively. For the young participant, the maximum values of the shown EMG signals were 0.902 and 0.812 mV for the right and left TA muscles, respectively, and 0.0588 and 0.0353 mV for the right and left MG muscles, respectively.

**Supplementary Figure S2. Cyclical EEG-EMG coherence of representative older and young participants during self-paced movements.** Coherence is calculated between C_z_ and the tibialis anterior (TA) and medial gastrocnemius (MG) muscles. For each muscle, the black and white patterns in the bottom row indicate the significant portions of the patterns in the top row.

**Supplementary Figure S3. Cortical distributions of significant coherence between EEG signals and EMG signals from the tibialis anterior (TA) and medial gastrocnemius (MG) muscles of representative older and young participants during self-paced movements.** C_z_ is circled. At each electrode location, the bar indicates the volume of significant coherence, measured in Hz multiplied by the percentage of movement cycle (Hz⋅%*_Movement Cycle_*). The scale of the vertical axis is the same for all distributions.

# Supplementary Tables

| **Parameters** | | **Interactions** | | |
| --- | --- | --- | --- | --- |
|  |  | **Aging×Type of Pacing** | **Aging×Side of Body** | **Type of Pacing×Side of Body** |
| Cycle Duration (Seconds) | *x* | *F*_1,87_ = 1.75, *p* = .189 | *F*_1,87_ = 7.45×10^-4^, *p* = .978 | *F*_1,87_ = 7.54×10^-4^, *p* = .978 |
|  | *s* | *F*_1,87_ = 0.118, *p* = .732 | *F*_1,87_ = 0.0224, *p* = .881 | *F*_1,87_ = 0.00425, *p* = .948 |
| Range of Motion  (Degrees) | *x* | *F*_1,87_ = 0.0808, *p* = .777 | *F*_1,87_ = 0.0903, *p* = .765 | *F*_1,87_ = 0.00864, *p* = .926 |
|  | *s* | *F*_1,87_ = 0.170, *p* = .681 | *F*_1,87_ = 0.0599, *p* = .807 | *F*_1,87_ = 679, *p* = .412 |
| *ϕ*  (Degrees) | *x* | *F*_1,43_ = 0.0724, *p* = .789 |  |  |
|  | *s* | *F*_1,43_ = 0.208, *p* = .651 |  |  |

**Supplementary Table S1. Interactions between the factors of ANOVA on the parameters of motor performance.** *x* and *s* indicate the intra-participant mean and standard deviation, respectively. *ϕ* is the relative phase that indicates the bilateral coordination of the limbs (*ϕ* = 180° for symmetrical coordination).

| **Interactions** | **Magnitude** | **Center Frequency** |
| --- | --- | --- |
| Aging×Type of Pacing | *F*_1,177_ = 0.00627, *p* = .937 | *F*_1,177_ = 0.0161, *p* = .899 |
| Aging×Muscle | *F*_1,177_ = 0.625, *p* = .430 | *F*_1,177_ = 0.444, *p* = .506 |
| Aging×Side of Body | *F*_1,177_ = 0.421, *p* = .517 | *F*_1,177_ = 0.00260, *p* = .959 |
| Type of Pacing×Muscle | *F*_1,177_ = 0.0245, *p* = .876 | *F*_1,177_ = 0.673, *p* = .413 |
| Type of Pacing×Side of Body | *F*_1,177_ = 0.137, *p* = .712 | *F*_1,177_ = 0.0245, *p* = .876 |
| Muscle×Side of Body | *F*_1,177_ = 0.0162, *p* = .899 | *F*_1,177_ = 0.0705, *p* = .791 |

**Supplementary Table S2. Interactions between the factors of 4-way ANOVA on the magnitude and frequency of significant EEG-EMG coherence.**

| **Parameter** | **Independent Variables** | | | |
| --- | --- | --- | --- | --- |
|  | **Aging** | **Type of Pacing** | **Muscle** | **Side of Body** |
| RMSD | *F*_1,113_ = 2.19, *p* = .142 | *F*_1,113_ = 1.10, *p* = .296 | *F*_1,113_ = 0.00176, *p* = .967 | *F*_1,113_ = 0.319, *p* = .573 |
| COD | *F*_1,113_ = 0.0155, *p* = .901 | *F*_1,113_ = 0.450, *p* = .504 | *F*_1,113_ = 0.0281, *p* = .867 | *F*_1,113_ = 0.673, *p* = .414 |
| *A* | *F*_1,113_ = 3.67, *p* = .0580 | *F*_1,113_ = 0.00585, *p* = .939 | *F*_1,113_ = 0.00694, *p* = .934 | *F*_1,113_ = 0.427, *p* = .515 |
| *σ*_RC_ | *F*_1,113_ = 3.19, *p* = .0768 | *F*_1,113_ = 8.62×10^-4^, *p* = .977 | *F*_1,113_ = 0.254, *p* = .615 | *F*_1,113_ = 0.186, *p* = .667 |
| *σ*_ML_ | ***F*_1,113_ = 4.03, *p* = .0471** | *F*_1,113_ = 2.04, *p* = .156 | *F*_1,113_ = 3.13, *p* = .0795 | *F*_1,113_ = 0.596, *p* = .442 |
| *μ*_RC_ | ***F*_1,113_ = 4.63, *p* = .0335** | *F*_1,113_ = 1.17, *p* = .282 | ***F*_1,113_ = 4.75, *p* = .0314** | *F*_1,113_ = 0.0483, *p* = .826 |
| *μ*_ML_ | *F*_1,113_ = 0.692, *p* = .407 | *F*_1,113_ = 0.446, *p* = .506 | *F*_1,113_ = 0.0313, *p* = .860 | *F*_1,113_ = 3.17, *p* = .0779 |

**Supplementary Table S3. Main effects of the factors of 4-way ANOVA on the parameters of fitted bivariate normal distributions.** Significant effects are indicated by the bold typeface. RMSD stands for root-mean-square deviation, and COD stands for coefficient of determination. *A*, *σ*, and *μ* are respectively the peak value, standard deviation, and mean of the fitted bivariate normal distributions. The subscripts, RC and ML, respectively indicate rostrocaudal and mediolateral directions.

| **Par.** | **Interactions** | | | | | |
| --- | --- | --- | --- | --- | --- | --- |
|  | **Aging**  **×Type of Pacing** | **Aging**  **×Muscle** | **Aging**  **×Side of Body** | **Type of Pacing**  **×Muscle** | **Type of Pacing**  **×Side of Body** | **Muscle**  **×Side of Body** |
| RMSD | F_1,113_ = 0.229,  *p* = .633 | F_1,113_ = 0.0618,  *p* = .804 | F_1,113_ = 0.193,  *p* = .661 | F_1,113_ = 2.13×10^-4^,  *p* = .988 | F_1,113_ = 0.0126,  *p* = .911 | F_1,113_ = 0.261,  *p* = .610 |
| COD | F_1,113_ = 0.358,  *p* = .551 | F_1,113_ = 1.91,  *p* = .170 | F_1,113_ = 0.101,  *p* = .751 | F_1,113_ = 3.35,  *p* = .0698 | F_1,113_ = 0.0200,  *p* = .888 | F_1,113_ = 1.47,  *p* = .228 |
| *A* | F_1,113_ = 0.00658,  *p* = .936 | F_1,113_ = 0.130,  *p* = .719 | F_1,113_ = 0.311,  *p* = .578 | F_1,113_ = 0.00398,  *p* = .950 | F_1,113_ = 0.0122,  *p* = .912 | F_1,113_ = 0.171,  *p* = .680 |
| *σ*_RC_ | F_1,113_ = 0.141,  *p* = .708 | F_1,113_ = 0.00691,  *p* = .934 | F_1,113_ = 0.625,  *p* = .431 | **F_1,113_ = 6.27,**  ***p* = .0137** | F_1,113_ = 0.293,  *p* = .589 | F_1,113_ = 1.92,  *p* = .169 |
| *σ*_ML_ | F_1,113_ = 0.0697,  *p* = .792 | F_1,113_ = 0.960,  *p* = .329 | F_1,113_ = 0.0277,  *p* = .868 | F_1,113_ = 0.0976,  *p* = .755 | F_1,113_ = 1.45,  *p* = .231 | F_1,113_ = 0.168,  *p* = .683 |
| *μ*_RC_ | F_1,113_ = 2.58,  *p* = .111 | F_1,113_ = 0.0275,  *p* = .869 | F_1,113_ = 0.350,  *p* = .556 | F_1,113_ = 0.749,  *p* = .389 | F_1,113_ = 0.110,  *p* = .741 | F_1,113_ = 0.0136,  *p* = .907 |
| *μ*_ML_ | F_1,113_ = 0.172,  *p* = .679 | F_1,113_ = 2.14,  *p* = .146 | F_1,113_ = 0.469,  *p* = .495 | F_1,113_ = 0.186,  *p* = .667 | F_1,113_ = 2.18,  *p* = .143 | F_1,113_ = 0.0759,  *p* = .784 |

**Supplementary Table S4. Interactions between the factors of 4-way ANOVA on the parameters (Par.) of fitted bivariate normal distributions.** Significant effects are indicated by the bold typeface. RMSD stands for root-mean-square deviation, and COD stands for coefficient of determination. A, σ, and μ are respectively the peak value, standard deviation, and mean of the fitted bivariate normal distributions. The subscripts, RC and ML, respectively indicate rostrocaudal and mediolateral directions.
